# Plant Natural Products Calycosin and Gallic Acid Synergistically Attenuate Neutrophil Infiltration and Subsequent Injury in Isoproterenol-Induced Myocardial Infarction: A Possible Role for Leukotriene B4 12-Hydroxydehydrogenase?

**DOI:** 10.1155/2015/434052

**Published:** 2015-07-21

**Authors:** Yuanyuan Cheng, Jia Zhao, Hung Fat Tse, X. Chris Le, Jianhui Rong

**Affiliations:** ^1^School of Chinese Medicine, Li Ka Shing Faculty of Medicine, The University of Hong Kong, 10 Sassoon Road, Pok Fu Lam, Hong Kong; ^2^Department of Medicine, Li Ka Shing Faculty of Medicine, The University of Hong Kong, 10 Sassoon Road, Pok Fu Lam, Hong Kong; ^3^Department of Laboratory Medicine and Pathology, Faculty of Medicine and Dentistry, 10-102 Clinical Sciences Building, University of Alberta, Edmonton, Alberta, Canada T6G 2G3

## Abstract

Leukotriene B4 12-hydroxydehydrogenase (LTB4DH) catalyzes the oxidation of proinflammatory LTB4 into less bioactive 12-oxo-LTB4. We recently discovered that LTB4DH was induced by two different natural products in combination. We previously isolated gallic acid from *Radix Paeoniae* through a bioactivity-guided fractionation procedure. The purpose of this study is to test the hypothesis that LTB4DH inducers may suppress neutrophil-mediated inflammation in myocardial infarction. We first isolated the active compound(s) from another plant, *Radix Astragali*, by the similar strategy. By evaluating LTB4DH induction, we identified calycosin and formononetin from *Radix Astragali* by HPLC-ESI-MS technique. We confirmed that gallic acid and commercial calycosin or formononetin could synergistically induce LTB4DH expression in HepG2 cells and human neutrophils. Moreover, calycosin and gallic acid attenuated the effects of LTB4 on the survival and chemotaxis of neutrophil cell culture. We further demonstrated that calycosin and gallic acid synergistically suppressed neutrophil infiltration and protected cardiac integrity in the isoproterenol-induced mice model of myocardial infarction. Calycosin and gallic acid dramatically suppressed isoproterenol-induced increase in myeloperoxidase (MPO) activity and malondialdehyde (MDA) level. Collectively, our results suggest that LTB4DH inducers (i.e., calycosin and gallic acid) may be a novel combined therapy for the treatment of neutrophil-mediated myocardial injury.

## 1. Introduction

Myocardial infarction is a leading cause of morbidity and mortality worldwide [1, 2]. Myocardial infarction triggers a sequence of inflammatory reactions involving the infiltration, activation, apoptosis, and clearance of neutrophils [3]. Thus, neutrophils play important roles in tissue damage, wound healing, cardiac remodeling, and scar formation [3–5]. Upon activation, neutrophils release reactive oxygen species, reactive nitrogen species, proteases, and possibly chemoattractant mediators for recruiting new inflammatory cells [3]. Interestingly, neutrophil depletion dramatically reduced infarct size in animal models of myocardial infarction [4, 5]. In addition, neutrophils produce various autacoids, such as thromboxane B and leukotriene B4 (LTB4), inducing vasoconstriction and platelet aggregation [6]. LTB4 is generated from membrane phospholipids by cytosolic phospholipase A2, 5-lipoxygenase, and leukotriene A4 (LTA4) hydrolase for recruiting and maintaining neutrophils [7–9]. Current anti-inflammatory therapies mainly target the formation and action of inflammatory mediators including LTB4 [10, 11]. Consequently, current LTB4-targeting drugs interrupt the progressive recruitment and sustained activation of neutrophils within infarcted myocardium [10, 12, 13].

Leukotriene B4 12-hydroxydehydrogenase (LTB4DH) is a multifunctional enzyme that catalyzes the oxidation of LTB4, the reduction of 15-oxo-prostaglandins (15-PGs), and the inactivation of 15-oxo-PGE and lipoxin A4 [14]. LTB4DH represents an endogenous mechanism for the control of LTB4 levels [15, 16]. Thereby LTB4DH may dampen neutrophil recruitment and promote the resolution of inflammation [17]. It is worth noting that several chemopreventive agents (e.g., dithiolethione) suppress inflammatory processes via inactivating LTB4 [15, 18]. On the other hand, LTB4DH is also induced as the fourth class of detoxification enzyme [19]. Collectively, pharmacological induction of LTB4DH expression may represent a novel strategy for the inhibition of LTB4-mediated inflammatory signals in infarcted myocardium.

Herbal medicines are well characterized for the inhibition of LTB4 biosynthesis [20–22]. Little is known about the potential of botanical compounds in the inactivation of LTB4 due to the limitation of the “one-drug-one-target” paradigm. Therefore, we developed a bias-free genome-wide biological response fingerprinting (BioReF) approach for the identification of target genes from the entire cellular genes in response to the complex mixture of plant natural products [23]. Thus, the target genes selected by BioReF may be responsive to two or multidrugs [24]. As a proof of principle, we previously identified LTB4DH as target gene for a well-documented poststroke rehabilitation formulation ISF-1 [23]. In fact, we discovered that LTB4DH was induced by the combination of two different herbal extracts (i.e.,* Radix Paeoniae Rubra* and* Radix Astragali*). We subsequently identified gallic acid from* Radix Paeoniae* for LTB4DH induction [24]. These results stimulated us to further identify the active compounds from* Radix Astragali* for LTB4DH induction within the context of myocardial infarction.

The present study was designed to test the hypothesis that LTB4DH inducers may suppress neutrophil-mediated inflammation in myocardial infarction. LTB4DH induction may directly decrease LTB4 level and thereby suppress LTB4-induced infiltration and survival of neutrophils in myocardial infarction. We isolated the active compounds from* Radix Astragali* for LTB4DH induction through a bioactivity-guided fractionation strategy. LTB4DH inducers were evaluated for the potential in the regulation of neutrophil chemotaxis and survival. Moreover, the cardioprotective effects of LTB4DH inducers were also examined in isoproterenol-induced mice model of myocardial infarction.

## 2. Materials and Methods

### 2.1. Antibodies and Biochemical Reagents

Rat monoclonal Ly6G antibody (RB6-8C5) was purchased from Abcam (Cambridge, MA, USA). Fluorescein isothiocyanate- (FITC-) labeled goat anti-rat IgG conjugate and RT-PCR reagents were purchased from Invitrogen (Carlsbad, CA, USA). Other biochemicals were purchased from Sigma-Aldrich (St. Louis, MO, USA) unless otherwise indicated. The oligonucleotide primers for LTB4DH and *β*-actin were obtained from Genome Research Centre, The University of Hong Kong.

### 2.2. Cell Culture and Drug Treatment

Human hepatocellular carcinoma cell line HepG2 was purchased from American Type Culture Collection (Rockville, MD, USA) and maintained in Eagle's minimum essential medium (MEM) containing 10% fetal bovine serum, 100 U/mL penicillin, and 100 *μ*g/mL streptomycin in a humidified incubator under 5% CO_2_ at 37°C. HepG2 cells were seeded in 6-well plate overnight and then treated with gallic acid alone or in combination with herbal fractions, calycosin and formononetin. After 24 h incubation, the total RNAs were extracted from the cells treated with indicated drugs and analyzed by RT-PCR technique.

### 2.3. Apparatus and Instruments

The HPLC system was equipped with two Waters 626 Model LC pumps, a Waters 717 Plus autosample injector, a Waters 996 Model photodiode array detector (DAD), a Waters 600S model system controller, and a gradient generator. Herbal profiles were obtained on a COSMOSIL PAQ-5 C18 column (250 mm × 4.6 mm i.d.). The elution was monitored by UV absorption within a spectrum ranging from 200 to 400 nm.* Radix Astragali* extracts were separated by a gradient mixture of acetonitrile as solvent A and 0.3% acetic acid water as solvent B in a binary gradient elution system. The gradient elution was performed as follows: 0–15 min, 10–25% A; 15–20 min, 25% A; 20–35 min, 25–34% A; 35–40 min, 34–45% A; 40–50 min, 45% A; 50–65 min, 45–65% A; 65–75 min, 65–80% A. The flow rate of mobile phase was 0.8 mL/min, and the column was maintained at 25°C.

### 2.4. Fractionation of* Radix Astragali* Extracts

**Figure 1:**
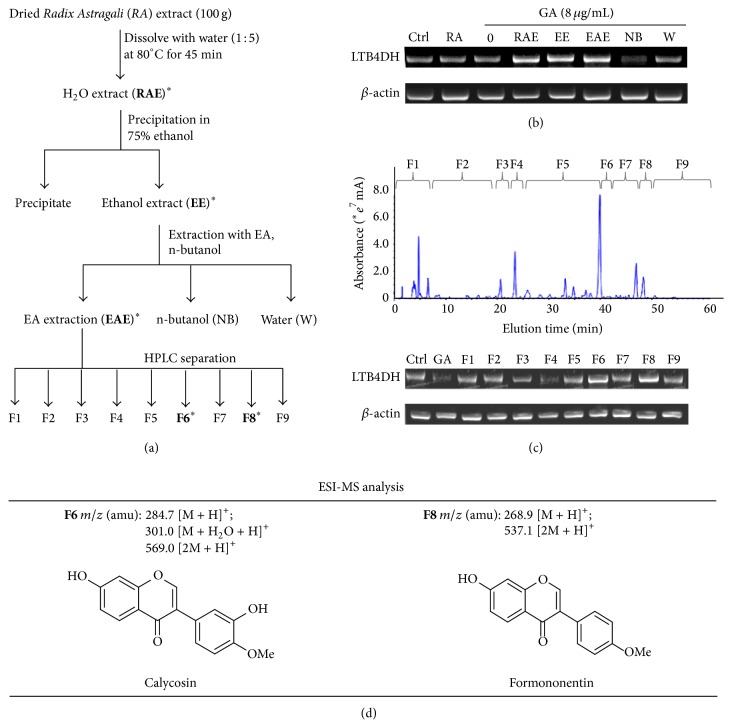
Bioactivity-guided isolation of the active compounds from* Radix Astragali* for LTB4DH induction. (a) Schematic illustration of the bioactivity-guided fractionation procedure. The active compounds were isolated from* Radix Astragali* extract through ethanol precipitation, extraction with organic solvents, and HPLC separation on a C18 column. The active fractions were highlighted with star (*∗*) and in bold. (b) RT-PCR detection of LTB4DH induction. The fractions in combination with gallic acid (GA) were assayed by semiquantitative RT-PCR for LTB4DH induction in HepG2 cells. (c) HPLC separation and RT-PCR detection of LTB4DH induction. Nine HPLC fractions in combination with gallic acid (GA) were assayed by semiquantitative RT-PCR for LTB4DH induction in HepG2 cells. The concentration of each fraction was normalized against the concentration of raw* Radix Astragali* (5.18 mg/mL). Representative HPLC profile and RT-PCR analysis were shown. (d) Chemical identification by HPLC-ESI-MS technique. The MS profiles of fraction 6 and 8 were shown. The structures of calycosin and formononetin were generated by ChemSketch software (http://www.acdlabs.com).

One hundred grams of dried* Radix Astragali* aqueous extract from Lianyungang Xingkang Biotechnology Co., Ltd. (Lianyungang, Jiangsu, China) was extracted with 0.5 liter of Millipore water at 80°C for 45 min (as Fraction-RAE in Figure 1). Following the centrifugation at 4000 rpm for 30 min, the supernatant was recovered and precipitated in 75% ethanol. The soluble materials (as Fraction-EE in Figure 1) were dried and redissolved in Millipore water for extraction with ethyl acetate (as Fraction-EAE in Figure 1) and n-butanol (as Fraction-NB in Figure 1). The water phase was defined as Fraction-W in Figure 1. Based on the results of RT-PCR detection, the active fraction was further separated into nine fractions by semipreparative HPLC as described in Section 2.3. The fractions generated from* Radix Astragali* extract were assayed for LTB4DH induction in combination with gallic acid as described in Section 2.2.

### 2.5. Reverse Transcription-Polymerase Chain Reaction (RT-PCR) Detection

The total RNAs were isolated from drug-treated cells with TRIzol reagent according to manufacturer's instructions (Invitrogen, CA, USA). cDNA templates were generated using SuperScript III reverse transcriptase and random hexamer primers (Thermo Scientific, Waltham, MA, USA). The expression of LTB4DH mRNA (NM_012212) was detected using sense primer, 5′-GAGCTTCAGGATGGTTCGTA-3′, and antisense primer, 5′-TCATGCTTTCACTATTGTCTTCC-3′, whereas *β*-actin mRNA (NM_001101) was detected as internal control using sense primer, 5′-GGCACCACACCTTCTACAATGA-3′, and antisense primer, 5′-GGAGTTGAAGGTAGTTTCGTGGA-3′. PCR amplifications were performed as follows: denaturation at 94°C for 3 min, 35 cycles of 94°C for 30 sec, 57°C for 30 sec, and 72°C for 1.15 min and extension at 72°C for 10 min. PCR products were analyzed by gel electrophoresis in 2% agarose containing Biotium GelRed DNA stain (Hayward, CA, USA) and visualized under UV light on Bio-Rad Gel Doc imaging system (Hercules, CA, USA).

### 2.6. Chemical Identification by Mass Spectrometry (MS)

The active HPLC fractions were analyzed by HPLC-MS on a Synergi Hydro-RP C18 (150 mm × 4.6 mm, 4 *μ*m) at the low rate of 0.7 mL/min under the control of an Agilent HPLC system equipped with a 1525 Separations Module and a 2998 DAD Unit. Mobile phase compositions were 0.3% (v/v) formic acid in water (A) and acetonitrile (B). Twenty microliters of sample was injected and eluted with the gradient mixture as follows: 0–15 min, 10–25% B; 15–20 min, 25% B; 20–35 min, 25–34% B; 35–40 min, 34–45% B; 40–50 min, 45% B; 50–65 min, 45–65% B; 65–75 min, 65–80% B. The eluents were analyzed on an AB Sciex triple quadrupole mass spectrometer 3200 QTRAP system (Framingham, MA, USA) equipped with an ESI-Turbo V source operating in positive ionization mode under the control of Analyst v1.4.2 data system (Applied Biosystems/MDS Sciex, Concord, ON, Canada). Mass spectrometry was operated under the conditions as follows: drying gas N2, 10 L/min; capillary voltage, 20 V; pressure of nebulizer, 30 psi; ion spray voltage, 4.5 kV; and capillary temperature, 325°C.

### 2.7. Isolation of Human Peripheral Blood Neutrophils

Human neutrophils were isolated from peripheral blood donated by healthy volunteers following a method described previously [25]. In brief, peripheral blood was subjected to dextran sedimentation. Erythrocytes were lysed in hypotonic solution. Neutrophils were recovered by the centrifugation in a gradient of Ficoll-Hypaque (GE Healthcare, Uppsala, Sweden). The cell viability was measured by trypan blue exclusion. Neutrophils were then resuspended in RPMI1640 medium for the use in the indicated assays.

### 2.8. Flow Cytometric Analysis of Cell Apoptosis

Neutrophils were treated with 10 *μ*M LTB4 alone or in combination with 30 *μ*M calycosin and 8 *μ*g/mL gallic acid in RPMI1640/10% FBS for 3, 6, 9, and 18 h. At the end of drug treatment, the cells were stained with Annexin V-FITC and PI according to manufacturer's instructions. Briefly, the cells were washed twice with ice-cold PBS and resuspended in 1x Binding Buffer at a concentration of 1 × 10^6^ cells/mL. Cell suspensions (1 × 10^5^ cells in 100 *μ*L) were incubated with 5 *μ*L of Annexin V-FITC and 10 *μ*L PI in a 5 mL culture tube at room temperature for 20 min. The stained cells were immediately analyzed on BD FACSVerse flow cytometry system (San Jose, CA, USA).

### 2.9. Assay of Chemotaxis

Freshly isolated human neutrophils were treated with 10 *μ*g/mL calycosin and 8 *μ*g/mL gallic acid individually or in combination. After 24 h incubation, the cell viability of neutrophils was found to be approximately 50% based on MTT assay. For the assay of chemotaxis, neutrophils were labelled by 5 *μ*M Calcein AM (Sigma, St. Louis, MO, USA). Labeled neutrophils (1.0 × 10^5^) were transferred onto the upper chamber of a Corning 24-well TransWell plate with 8 *μ*m pore polycarbonate membrane insert (Tewksbury, MA, USA). Cell culture medium containing LTB4 (0, 10, or 100 nM) was placed in the lower chamber. After 3 h incubation, TransWell plate was centrifuged at 200 ×g for 5 min. The cells in the lower chamber were incubated with the cell dissociation solution for 30 min. The fluorescence was measured on a fluorescence microplate reader at excitation/emission wavelengths of 485/530 nm (Molecular Devices, Sunnyvale, CA, USA).

### 2.10. Isoproterenol-Induced Mouse Model of Myocardial Infarction

The protocol for the experiments on isoproterenol-induced mouse model of myocardial infarction was approved by the Committee on the Use of Live Animals in Teaching and Research (CULATR), The University of Hong Kong. Thirty male C57BL/6 mice (6–8 weeks old, 20–22 g) were obtained from Laboratory Animal Unit, The University of Hong Kong. The mice were allowed free access to standard rodent chow and clean drinking water. For drug treatment, the mice were randomly divided into the five treatment groups (*n* = 6) as follows: Control group, receiving saline and vehicle (0.5 mL/day) via i.p. injection for 3 days; ISO group, receiving saline (0.5 mL/day) via i.p. injection for the first day and isoproterenol (100 mg/kg/day, 200 *μ*L) via s.c. injection for another 2 consecutive days; ISO + GA group, receiving gallic acid (8 mg/kg/day, 500 *μ*L) via i.p. injection for 3 consecutive days and isoproterenol (100 mg/kg/day, 200 *μ*L) via s.c. injection on day 2 and day 3; ISO + CA group, receiving calycosin (40 mg/kg/day, 500 *μ*L) via i.p. injection for 3 consecutive days and isoproterenol (100 mg/kg/day, 200 *μ*L) via s.c. injection on day 2 and day 3; ISO + CA + GA group, receiving calycosin (40 mg/kg/day) and gallic acid (8 mg/kg/day) in 500 *μ*L via i.p. injection for 3 consecutive days and isoproterenol (100 mg/kg/day, 200 *μ*L) via s.c. injection on day 2 and day 3. Isoproterenol was dissolved in 0.9% saline, whereas calycosin and gallic acid were dissolved in 5% ethanol in 0.9% saline. Isoproterenol was injected 10 min after the injection of calycosin and/or gallic acid as previously described and dosed [26].

### 2.11. Histopathological Evaluation of Cardioprotection

Half of the animals from each interventional group were euthanized by injecting pentobarbital (200 mg/kg, i.p.) and fixed by perfusing 4% paraformaldehyde. The heart tissues were embedded in paraffin and dissected into 5 *μ*m tissue sections. Cardiac sections were stained with hematoxylin and eosin (H&E) stain and examined for gross myocyte injury under light microscope as previously described [27].

### 2.12. Immunohistochemical Studies of Neutrophils

Cardiac sections were boiled in citrate buffer (pH = 6) in microwave oven for 15 min with a 5 min interval. Following blocking in 5% goat serum for 1 h, cardiac sections were probed with primary antibody against murine Ly6G (1 : 50) at 4°C overnight. After 5 washes with PBS, cardiac sections were subsequently incubated with FITC-conjugated anti-rat IgG secondary antibody (1 : 200) at room temperature for 1.5 h. The fluorescent images were acquired on a LSM 700 laser scanning microscope (Carl Zeiss, Jena, Germany).

### 2.13. Assay of Myeloperoxidase (MPO) and Malondialdehyde (MDA)

The frozen hearts were homogenized in 1 mL PBS (pH 6.0) supplemented with 0.5% hexadecyltrimethylammonium hydroxide and centrifuged at 12,000 rpm at 4°C for 20 min. The supernatants were recovered for the determination of MPO activity and MDA level. The protein concentration in the supernatants was determined with a Bio-Rad protein assay kit (Hercules, CA, USA) according to manufacturer's instructions. The MPO activity was detected by using a commercial MPO detection kit (Jiancheng Bioengineering Institute, Nanjing, China) according to manufacturer's instructions. One unit of MPO activity is defined as degrading 1 *μ*mol of hydrogen peroxide at 37°C. The MPO activity was determined by measuring the absorbance at 460 nm on a PerkinElmer UV/VIS spectrophotometer (Waltham, MA, USA) and expressed as units per gram of total proteins (U/g proteins).

MDA activity was measured by thiobarbituric acid assay kit (Jiancheng Bioengineering Institute, Nanjing, China) according to manufacturer's instructions. Briefly, the supernatant was incubated with thiobarbituric acid. The absorbance at 532 nm was determined on a PerkinElmer UV/VIS spectrophotometer (Waltham, MA, USA). The MDA level was calculated in the format of nmol/mg proteins.

### 2.14. Statistical Analysis

All results were expressed as the mean ± SD. Differences between groups were analyzed by one-way analysis of variance (ANOVA) followed by Tukey's test or two-tail paired Student's *t*-test with GraphPad Prism 5 software (La Jolla, CA, USA). The *p* values less than 0.05 were considered to be statistically significant.

## 3. Results

### 3.1. Bioactivity-Guided Isolation of LTB4DH Inducers from* Radix Astragali* Extract

*Radix Astragali* extract was fractionated into different fractions following the bioactivity-guided fractionation procedure as illustrated in Figure 1(a). Based on our previous studies, LTB4DH expression was induced by the combination of the active compounds from* Radix Astragali* and gallic acid identified from* Radix Paeoniae* [24]. Thus, we assayed all* Radix Astragali-*derived fractions in the presence of 8 *μ*g/mL gallic acid. As shown in Figure 1(b), neither* Radix Astragali* extract (RA) nor gallic acid (GA) induced LTB4DH expression. In combination with 8 *μ*g/mL gallic acid, interestingly, the initial H_2_O extract (RAE), ethanol extract (EE), and ethyl acetate extraction (EAE) were found to markedly induce LTB4DH mRNA expression. We subsequently separated the Fraction-EAE into 9 fractions by RP-HPLC on a C18 column. All HPLC fractions were assayed in combination with 8 *μ*g/mL gallic acid for LTB4DH induction in HepG2 cell line. Based on RT-PCR detection (Figure 1(c)), two fractions (i.e., Fraction-6 and Fraction-8) were found to be active in the induction of LTB4DH mRNA. Fraction-6 and Fraction-8 appeared to be single peak in HPLC profiles under different conditions. Thus, Fraction-6 and Fraction-8 were further characterized on a HPLC-MS system. As shown in Figure 1(d), three major signals, 284.7 [M + H]^+^, 301.0 [M + H_2_O + H]^+^, and 569 [2M + H]^+^, were detected in the ESI-MS spectrum of Fraction-6 in positive ion mode. Interestingly, the MS profile of Fraction-6 matches that of calycosin. On the other hand, Fraction-8 showed two major positive ions including 268.9 [M + H]^+^ and 537.1 [2M + H]^+^ matching the MS profile of formononetin.

### 3.2. Verification of LTB4DH Induction by Commercial Calycosin and Formononetin in Neutrophils

**Figure 2:**
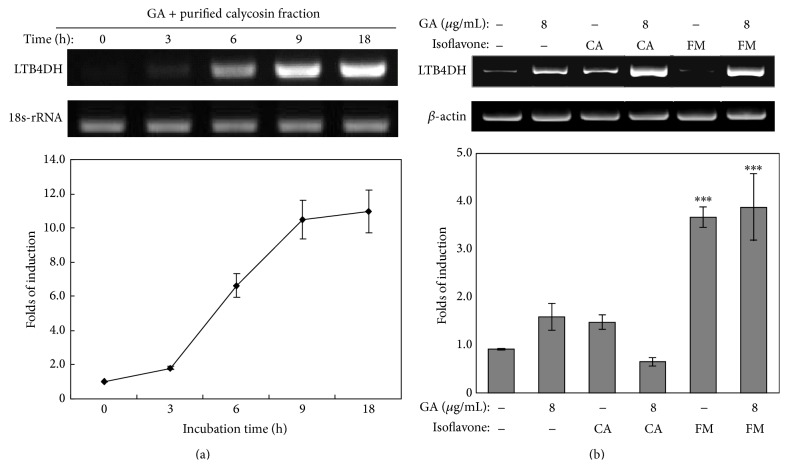
Verification of purified calycosin fraction and commercial compounds (i.e., calycosin and formononetin) for LTB4DH induction in human neutrophils. (a) LTB4DH induction by purified calycosin fraction and gallic acid (GA) in combination. Human neutrophils were freshly isolated and treated with purified calycosin fraction (28 *μ*g/mL) and gallic acid (GA) (8 *μ*g/mL) in combination for indicated time points. (b) LTB4DH induction by commercial compounds (i.e., calycosin (CA) and formononetin (FM)) and gallic acid (GA) in combination. Human neutrophils were treated with calycosin (10 *μ*g/mL), formononetin (10 *μ*g/mL), and gallic acid (8 *μ*g/mL), alone or in combination, for 24 h. LTB4DH expression was assayed by RT-PCR. The mean values of three independent experiments were shown. Significance analysis was performed by two-tail paired Student's* t*-test. ^*∗∗∗*^ *p* < 0.001 (sample versus untreated control).

We first examined the kinetics of LTB4DH expression in response to the stimulation with the drugs in combination for different times (i.e., 0, 3, 6, 9, and 18 h). The purified calycosin fraction (28 *μ*g/mL) and commercial gallic acid (8 *μ*g/mL) in combination induced the expression of LTB4DH mRNA in a time-dependent manner (Figure 2(a)). LTB4DH mRNA was detectable after 6 h treatment in our assay system.

To confirm the MS identification of the active compounds, we treated human neutrophils with commercial calycosin (10 *μ*g/mL), formononetin (10 *μ*g/mL), and gallic acid (8 *μ*g/mL), individually or in combination, for 24 h. Total RNAs were extracted from the drug-treated cells and analyzed by RT-PCR technique for LTB4DH mRNA expression. As shown in Figure 2(b), commercial calycosin and formononetin in combination with gallic acid significantly induced LTB4DH expression, whereas calycosin, formononetin, and gallic acid alone showed much weaker activity.

### 3.3. Inhibition of LTB4-Induced Neutrophil Survival

**Figure 3:**
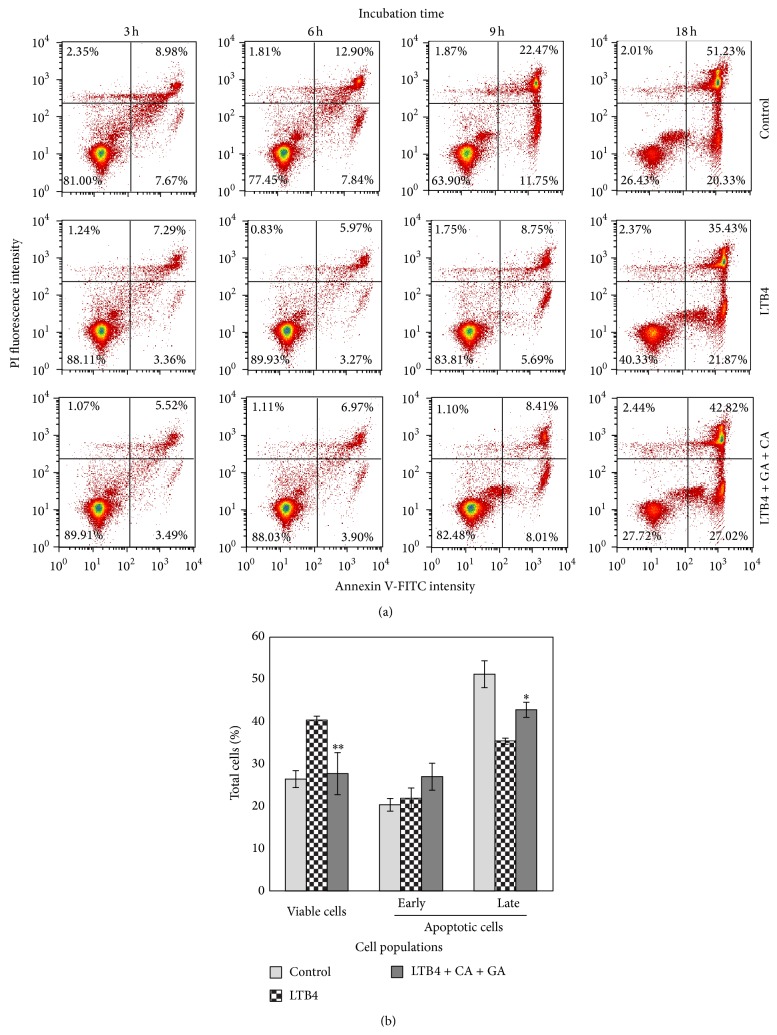
Suppression of LTB4-induced neutrophils survival by calycosin and gallic acid. (a) Human neutrophils were freshly prepared and were treated with 100 nM LTB4 alone or in combination with calycosin (CA) (10 *μ*g/mL) and gallic acid (GA) (8 *μ*g/mL) for the indicated times. The apoptotic cells were detected by staining Annexin V-FITC/PI and profiled by flow cytometry. The apoptotic cells were characterized into early apoptotic population (lower right panel) and later apoptotic population (upper right panel). (b) Quantitation of neutrophil populations (viable, early apoptotic, and late apoptotic).

To examine the biological impacts of LTB4DH inducers, we first investigated the effect of calycosin and gallic acid in combination on LTB4-mediated cell survival signals. We treated human neutrophils by LTB4 alone or in combination with calycosin and gallic acid for different times. The cells were subsequently stained with FITC-Annex-V and PI and analyzed by flow cytometry. Compared with untreated controls, LTB4 effectively prevented neutrophils from spontaneous apoptosis. In the presence of LTB4, early apoptotic neutrophils ranged from 3.36% to 5.69% in the first 9 hours, whereas untreated control neutrophils underwent progressive apoptosis ranging from 7.67% to 11.75%. Importantly, calycosin and gallic acid in combination effectively diminished the prosurvival effect of LTB4 on the cell viability of neutrophils (Figure 3). In particular, after 18 h cotreatment with LTB4, calycosin, and gallic acid, the number of viable neutrophils was dramatically decreased from 40.3% to 27.7%, highly comparable with 26.4% for untreated neutrophils.

### 3.4. Synergistic Inhibition of Neutrophil Chemotaxis by Calycosin and Gallic Acid

**Figure 4:**
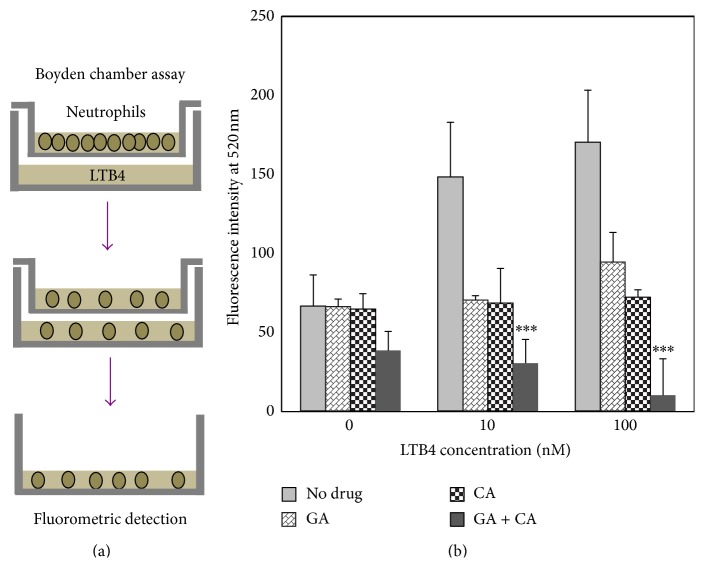
Synergistic inhibitory effect of gallic acid and calycosin on LTB4-induced chemotaxis of neutrophils. (a) Schematic representation of neutrophil chemotaxis assay. (b) Quantitation of neutrophil chemotaxis. Human neutrophils were Isolated and treated with gallic acid and calycosin, individually or in combination, at 37°C for 24 h. At the end of treatment, neutrophils were loaded with fluorescent probe Calcein AM. Chemotaxis of neutrophils from the upper chamber towards LTB4 in lower chamber was assayed as outlined in (a). Migrated neutrophils were quantitated by measuring the fluorescence intensity on a Zeiss fluorescence spectroscopy (Jena, Germany). Data were expressed as the mean ± SD. Statistical analysis was performed using one-way ANOVA followed by Tukey's test. ^*∗∗∗*^ *p* < 0.001 (Drugs + LTB versus LTB4).

We subsequently examined the effect of calycosin and gallic acid in combination on LTB4-induced neutrophil chemotaxis. We treated human neutrophils with calycosin and gallic acid, individually or in combination, for 24 h. The neutrophils were seeded in the upper chamber of TransWell plate and allowed to migrate through 8 *μ*m pore polycarbonate membrane towards the cell culture medium supplemented with LTB4 (0, 10, and 100 nM) over a period of 4 h (Figure 4(a)). The neutrophils were recovered from the lower chamber of TransWell plate and quantified by measuring the fluorescence from preloaded Calcein AM. As shown in Figure 4(b), calycosin and gallic acid in combination showed strongest inhibitory effect on LTB4-induced chemotaxis of neutrophils, although calycosin and gallic acid alone also prevented the chemotaxis of neutrophils in response to LTB4 stimulation.

### 3.5. Synergistic Cardioprotective Effects of Calycosin and Gallic Acid against Isoproterenol-Induced Myocardial Infarction

**Figure 5:**
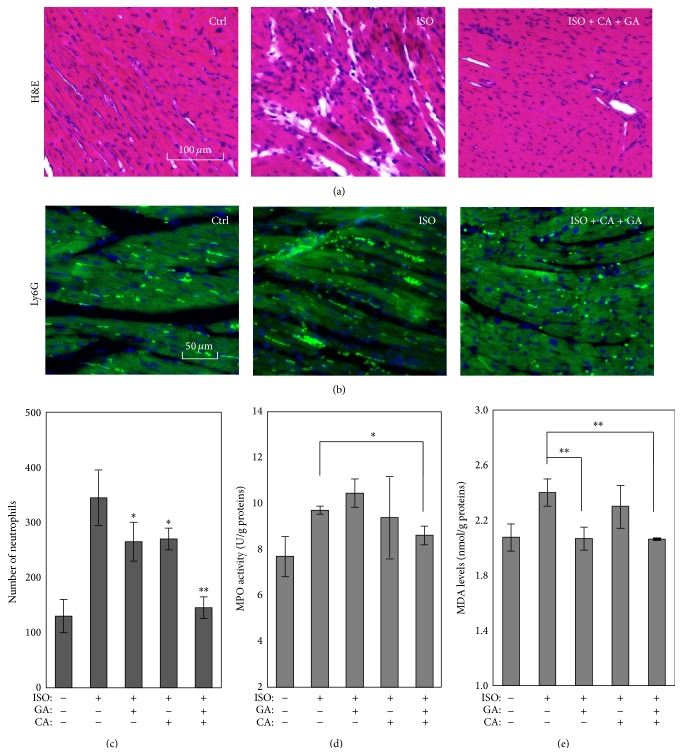
*In vivo *cardioprotective effects of calycosin and gallic acid in combination. (a) H&E histopathological staining of mouse heart tissues. Five groups (*n* = 3) of C57BL/6 mice were treated with vehicle and isoproterenol (ISO); ISO + GA; ISO + CA and ISO + GA + CA. The cardiac tissues were stained with H&E. Representative images were selected from five groups. (b) Immunohistofluorescence staining of neutrophil biomarker Ly6G in mouse heart tissues. Following the treatment as described in (a), the heart tissues were probed with Ly6G antibody and subsequently detected with Alexa Fluor 488-labeled secondary antibody. The cell nuclei were stained with DAPI. The images were captured under a Zeiss fluorescence microscope. Representative images were selected from five groups. (c) Quantitation of neutrophil infiltration. Ly6G-positive cells were counted under a Zeiss fluorescence microscope. The mean numbers of neutrophils were shown and analyzed by one-way ANOVA followed by Tukey's test. ^*∗*^ *p* < 0.05 (Drugs + ISO versus ISO group). (d) MPO activity in the cardiac tissues. Following the treatment as described in (a), the heart tissues were homogenized for the assay of MPO activity. The values represent the mean ± SD. ^*∗*^ *p* < 0.05 (Drugs + ISO versus ISO group). (e) MDA levels in the cardiac tissues. Following the treatment as described in (a), the heart tissues were homogenized for the determination of MDA levels. The values represent the mean ± SD. ^*∗*^ *p* < 0.05; ^*∗∗*^ *p* < 0.01 (Drugs + ISO versus ISO group).

To further determine the cardioprotective effect of LTB4DH inducers, we treat mice with calycosin and gallic acid, individually or in combination, for three consecutive days. On day 2, isoproterenol was injected once a day for two consecutive days to induce myocardial infarction in mice. At the end of drug treatment, heart tissues were stained with H&E stain. Compared with untreated controls, isoproterenol induced the focal or diffused infiltration of lymphocytes, plasma cells, and polymorphonuclear leukocytes, thereby indicating myocytolysis. Calycosin and gallic acid alone failed to attenuate isoproterenol-induced myocytolysis. Remarkably, calycosin and gallic acid synergistically attenuated isoproterenol-induced alteration of cardiac morphology (Figure 5(a)).

To examine the* in vivo* effect of calycosin and gallic acid on neutrophils infiltration, we probed the cardiac tissues with specific antibody against neutrophil biomarker Ly6G and detected by FITC-labeled anti-rat IgG. The neutrophils were detected and counted under a fluorescence microscope. As shown in Figures 5(b) and 5(c), calycosin and gallic acid synergistically eliminated the effect of isoproterenol on neutrophil infiltration, although calycosin and gallic acid alone also showed impressive inhibitory effect on isoproterenol-induced neutrophil infiltration.

To further verify the* in vivo* effect of calycosin and gallic acid on neutrophils infiltration, we determined the MPO activity in the cardiac tissues after the treatments with calycosin, gallic acid, and isoproterenol. We found that calycosin and gallic acid in combination significantly prevented isoproterenol-induced increase in the MPO activity, whereas neither calycosin nor gallic acid affected the MPO activity in isoproterenol-treatment animals (Figure 5(d)).

We also examined the effect of calycosin and gallic acid on lipid peroxidation by measuring MDA levels [28]. We determined the levels of MDA in heart tissues by a MDA detection kit. As shown in Figure 5(e), gallic acid alone and in combination with calycosin effectively reduced isoproterenol-induced increase in the formation of MDA. In contrast, calycosin alone did not show any inhibitory effect on isoproterenol-induced increase in the formation of MDA.

## 4. Discussion

Most of the drugs in the market are basically developed according to the “one-drug-one-target” paradigm in drug discovery [29]. We recently discovered that LTB4DH expression was induced by the coordination between two active compounds but not either of two compounds alone in human cells by a genome-wide biological response fingerprinting (BioReF) approach [23, 24]. With the knowledge of human genome and proteome, the network approach helped the design of multitarget drugs [30, 31]. Based on our previous discoveries, on the other hand, we proposed a new concept of “multidrugs on one target.” We believe that future drug development may focus on the synergy of multiple common compounds against one single pharmacological target. By targeting LTB4DH, for example, we previously identified gallic acid as one of the active compounds from the herb* Radix Paeoniae Rubra* through a bioactivity-guided fractionation strategy [24]. In the present study, we searched for the active compounds from the other herb* Radix Astragali*. By assaying LTB4DH induction in human HepG2 cells, we isolated two* Radix Astragali*-derived fractions, namely, Fraction-6 and Fraction-8, as the active fractions for inducing LTB4DH expression in combination with gallic acid. HPLC-MS analysis revealed that Fraction-6 and Fraction-8 showed identical MS profiles to calycosin and formononetin, respectively. One of the key findings from the current study was that calycosin and gallic acid in combination could effectively attenuate isoproterenol-induced myocardial injury in mice.

Neutrophils are known to be activated in the infarcted myocardium to release free radicals, inflammatory cytokines, and mediators such as superoxide ions, nitric oxide, TNF-*α*, LTB4, and PGE2 [32, 33]. Consequently, infiltrating neutrophils may damage surrounding myocytes and endothelial cells via close contacts or release of inflammatory mediators [3, 34]. As the key example of proinflammatory mediators, LTB4 is well known to regulate the infiltration and survival of neutrophils in response to myocardial injury [9, 35, 36]. Owing to the action of LTB4DH on LTB4, we postulated that LTB4DH induction might possibly attenuate neutrophil-mediated myocardial injury via inactivating LTB4. The identification of LTB4DH inducers should allow the pharmacological interruption of the recruitment and sustained activation of neutrophils in myocardial infarction via inactivating LTB4.

In the present study, we first demonstrated that calycosin and gallic acid in combination inhibited LTB4-induced survival and chemotaxis of human neutrophils* in vitro*. We also investigated the effects of two LTB4DH inducers (i.e., calycosin and gallic acid) on neutrophil infiltration and subsequent myocardial injury in isoproterenol-induced mouse model of myocardial infarction. Following the drug treatment, the cardiac tissues were stained by H&E and antibody against neutrophil biomarker Ly6G [37]. H&E staining revealed that calycosin and gallic acid in combination preserved the cardiac morphology against isoproterenol-induced disruption. Fluorescent detection of Ly6G binding revealed that calycosin and gallic acid in combination significantly reduced the infiltration of neutrophils following isoproterenol-induced myocardial injury. MPO activity is widely used to indicate the infiltration and activation of neutrophils in myocardial infarction [38]. We indeed found that calycosin and gallic acid in combination significantly reduced isoproterenol-induced MPO activity in cardiac tissues. On the other hand, the MDA level serves a common indicator for isoproterenol-induced lipid peroxidation [28, 39]. In our experiments, calycosin and gallic acid in combination significantly attenuated the effect of isoproterenol on MDA formation, whereas gallic acid also showed similar inhibition on MDA formation. These results strongly suggested two LTB4DH inducers (i.e., calycosin and gallic acid) could be used to restrict neutrophil infiltration and subsequent myocardial injury in isoproterenol-induced mouse model of myocardial infarction.

## 5. Conclusion

**Figure 6:**
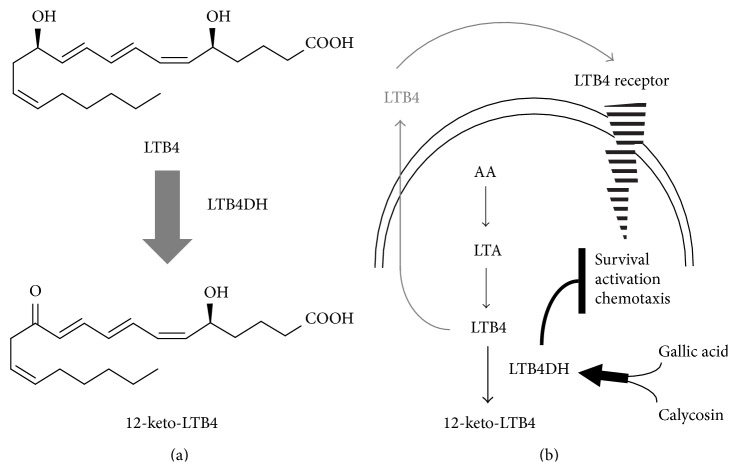
Potential impacts of LTB4DH induction on the cellular level and activities of LTB4 in neutrophils. (a) Inactivation of LTB4 by LTB4DH. (b) LTB4DH induction and subsequent effect on LTB4-mediated cellular signaling pathways.

In summary, the present study reported rapid identification of calycosin and formononetin from* Radix Astragali* extract as LTB4DH inducer through a bioactivity-guided fractionation procedure. Calycosin and gallic acid in combination not only synergistically induced LTB4DH expression in neutrophils but also attenuated the effects of LTB4 on the survival and chemotaxis of neutrophils (Figure 6). Importantly, calycosin and gallic acid in combination effectively suppressed neutrophils infiltration and myocardial injury in isoproterenol-induced mice model of myocardial infract. Thus, the present study will promote the pharmacological use of multiple common compounds in combination in the treatment of complex myocardial infarction.
